# Effects of a Low Dose of Orally Administered Creatine Monohydrate on Post-Fatigue Muscle Power in Young Soccer Players

**DOI:** 10.3390/nu16091324

**Published:** 2024-04-28

**Authors:** Álvaro Huerta Ojeda, Emilio Jofré-Saldía, Maximiliano Torres-Banduc, Sergio Galdames Maliqueo, Guillermo Barahona-Fuentes, Carlos Cofré Acevedo, Gabriela Lizana Romero, Regina de Villa Garduño, Gerardo Riquelme Vera, Pablo Vera Paredes, Benjamín Barrios Ávalos, Tatiane Morales Serey, María-Mercedes Yeomans-Cabrera, Carlos Jorquera-Aguilera

**Affiliations:** 1 Núcleo de Investigación en Salud, Actividad Física y Deporte ISAFYD, Universidad de Las Américas, Viña del Mar 2531098, Chile; mtorres@udla.cl (M.T.-B.); danielbarahonaf@gmail.com (G.B.-F.); carloscofre01@gmail.com (C.C.A.); glizana@udla.cl (G.L.R.); nutricionistareginadevilla@gmail.com (R.d.V.G.); gera.rv@hotmail.com (G.R.V.); pabloveraparedes@gmail.com (P.V.P.); benjamin_1495@hotmail.com (B.B.Á.); tmoraless@udla.cl (T.M.S.); 2 Escuela de Ciencias de la Actividad Física, el Deporte y la Salud, Universidad de Santiago de Chile USACH, Santiago 7550000, Chile; emilio.jofre.s@usach.cl; 3 Facultad Ciencias de la Actividad Física y del Deporte, Universidad de Playa Ancha de Ciencias de la Educación, Valparaíso 2340000, Chile; sergio.galdames@upla.cl; 4 Faculty of Education and Social Sciences, Universidad Andres Bello, Viña del Mar 2520000, Chile; 5 Facultad de Salud y Ciencias Sociales, Universidad de Las Américas, Viña del Mar 2531098, Chile; mmyeomans@outlook.com; 6 Facultad de Ciencias, Escuela de Nutrición y Dietética, Universidad Mayor, Santiago 8580745, Chile; carlos.jorquera@mayor.cl

**Keywords:** ergogenic aid, fatigue, athletic performance, soccer

## Abstract

The use of creatine monohydrate (Cr) in professional soccer is widely documented. However, the effect of low doses of Cr on the physical performance of young soccer players is unknown. This study determined the effect of a low dose of orally administered Cr on muscle power after acute intra-session fatigue in young soccer players. Twenty-eight young soccer players (mean age = 17.1 ± 0.9 years) were randomly assigned to either a Cr (n = 14, 0.3 g·kg^−1^·day^−1^ for 14 days) or placebo group (n = 14), using a two-group matched, double-blind, placebo-controlled design. Before and after supplementation, participants performed 21 repetitions of 30 m (fatigue induction), and then, to measure muscle power, they performed four repetitions in half back squat (HBS) at 65% of 1RM. Statistical analysis included a two-factor ANOVA (*p* ˂ 0.05). Bar velocity at HBS, time: *p* = 0.0006, ${ŋ}_{p}^{2}$ = 0.22; group: *p* = 0.0431, ${ŋ}_{p}^{2}$ = 0.12, time × group *p* = 0.0744, ${ŋ}_{p}^{2}$ = 0.02. Power at HBS, time: *p* = 0.0006, ${ŋ}_{p}^{2}$ = 0.12; group: *p* = 0.16, ${ŋ}_{p}^{2}$ = 0.06, time × group: *p* = 0.17, ${ŋ}_{p}^{2}$ = 0.009. At the end of the study, it was found that, after the induction of acute intra-session fatigue, a low dose of Cr administered orally increases muscle power in young soccer players.

## 1. Introduction

Soccer is characterized by intermittent high-intensity efforts (running, jumping, and sprinting) combined with low-to-moderate intensity actions (walking and jogging) and periods of limited activity or stationary rest [1]. In this scenario, muscular strength and power, along with their related components, such as speed and acceleration, have emerged as critical elements to be addressed during the training of soccer players at all levels [2]. In this sense, an efficient physical preparation, oriented toward the development of muscular strength and its adequate transfer to power on the playing field, will allow for a better performance in the activities of this sport [3]. In this context, the squat is one of the most widely used exercises for strength development in professional and semiprofessional soccer players [2], and quantifying squat load based on movement velocity generates significant improvements in the relative and absolute power of players on the field [4]. At the same time, concurrent training has emerged as another method that, in addition to improving neuromuscular function, allows for the joint development of aerobic and muscular strength parameters [5]. Therefore, in modern soccer, for both professional and youth soccer players, the development and control of muscular strength is essential to achieve optimal physical performance.

During training, especially during matches, the constant high-intensity repetitions typical of soccer induce physical fatigue. This condition can be evident during and after matches [3] and within the training (acute intra-session fatigue). Precisely, fatigue corresponds to any decrease in muscular performance [6], a condition that, from an intrinsic point of view, is affected by a combination of factors ranging from alterations in the central nervous system, muscle cells, and energy production [7]. Along these lines, factors such as match result, quality of the opponent, match location, and playing surface influence the occurrence and level of fatigue experienced by the players [3] and, consequently, in the physical performance they achieve [8,9]. As a solution to these problems, different strategies have been developed to achieve an efficient recovery of soccer players of all categories. These range from quantifying recovery time after the match [10] and training [11] to using ergogenic aids to delay the onset of fatigue and increase physical performance [12].

Ergogenic aids—which include mechanical aspects such as sports clothing and/or footwear, psychological aspects such as visualization, physiological aspects such as growth hormone, and nutritional aspects such as the use of supplements—are, as a whole, essential to improve physical performance [13], recovery after exercise, and injury prevention [14]. Among the ergogenic nutritional aids, creatine monohydrate (Cr) stands out as one of the substances most valued by athletes of all levels [15,16], mainly for its effectiveness in increasing muscle strength and power, as well as its ability to improve performance in high-intensity activities, e.g., sprints and/or exercises requiring short bursts of energy (anaerobic metabolism) [14,17,18,19]. In this context, the scientific literature suggests that a dose of 0.3 g Cr per kilogram of body weight per day (0.3 g·kg^−1^·day^−1^) for one week, followed by a maintenance dose of 5 g per day, is sufficient to observe significant improvements in physical performance [19]. Likewise, using Cr has shown favorable results on anaerobic metabolism in professional and youth soccer players [20,21]. For example, Mielgo-Ayuso et al. [21] found that an effective dose of Cr supplementation should include a loading dose of 20–30 g·day^−1^, divided over 3–4 times a day, ingested for 6–7 days, followed by 5 g·day^−1^ for nine weeks or a low dose of 3 mg·kg^−1^·day^−1^ for 14 days or more [21]. On the other hand, Yañez-Silva et al. [22] evidenced an improvement in muscle power with a low dose of Cr in the short term. However, these authors did not induce fatigue before the evaluation [22]. Indeed, Cr and phosphagen, catalyzed by the enzyme creatine kinase (CK), play a fundamental role in the supply of energy in alactic anaerobic metabolism, mainly due to their ability to synthesize adenosine triphosphate (ATP) from phosphocreatine (PCr) and adenosine diphosphate (ADP) [23].

Despite these recommendations, the current literature needs to clarify the impact of a low dose of Cr, following the induction of acute fatigue during the session, on muscle power in half back squat (HBS) [20,21], generating an essential gap in our knowledge. In this scenario, the present study seeks to reduce the knowledge gap by determining the effect of a low dose of orally administered Cr on muscle power after acute intra-session fatigue in young soccer players. This study’s hypothesis indicates that 0.3 g·kg^−1^·day^−1^ Cr for 14 days increases execution velocity and power generated in HBS after the induction of acute intra-session fatigue. As a secondary objective, force, rate of force development (RFD) in HBS, fatigue index (FI), and total time in the repeated sprint ability (RSA) test were determined.

## 2. Materials and Methods

### 2.1. Design

**Figure 1:**
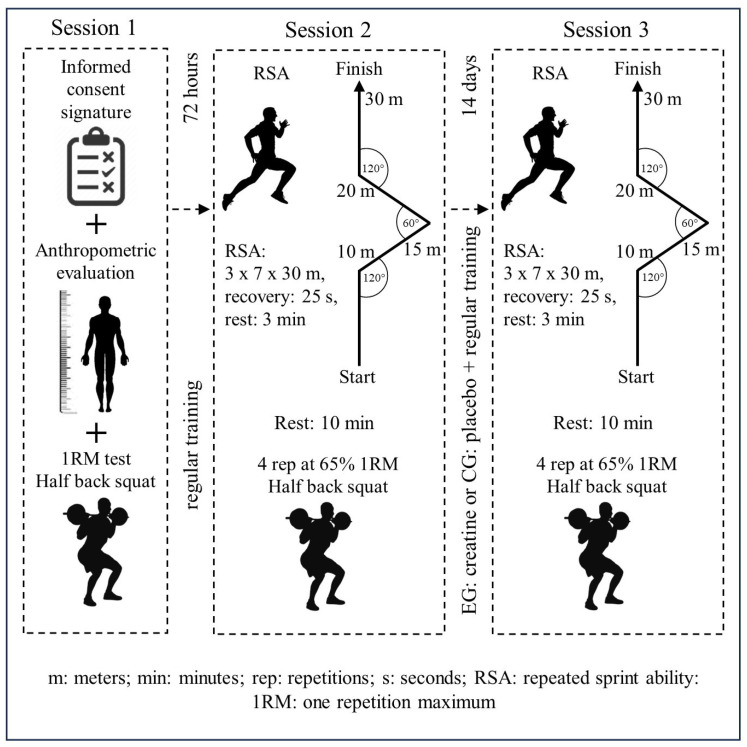
Research design.

The research considered a two-group matched, double-blind, placebo-controlled design [24]. The intervention included 28 young soccer players (17.1 ± 0.9 years), who were distributed in the experimental group (EG, n = 14) and control group (CG, n = 14). During the 14 days of supplementation, both groups maintained regular training, corresponding to 90 min (min) of training with technical, tactical, and physical orientation six days per week. The variables used for sample matching were age and lower extremity strength (1RM in HBS). The EG received an intake of 0.3 g of Cr powder per kilogram of body mass (0.3 g·kg^−1^) for 14 days [19], while the CG had an intake of 0.3 g of maltodextrin per kg of body mass in the same period. Before and after the intervention, to induce fatigue similar to that experienced during a soccer match, participants were asked to perform 21 repetitions (reps) of 30 m, divided into three sets of 7 reps each, with 25 s of passive recovery between repetitions and 3 min of rest between sets (21 RSA: 3 × 7 × 30 m, recovery 25 s, rest 3 min). After the RSA test, participants were asked to perform four repetitions in HBS with a load equivalent to 65% of 1RM. Specifically, the HBS outcomes were used to observe the possible effects of Cr (Figure 1).

### 2.2. Participants

Twenty-eight young soccer players from the Everton Club of Viña del Mar, Chile, volunteered to participate in the study. The sample was classified as “Trained” [25], non-probabilistic, and distributed by sample matching (EG n = 14 and CG n = 14). Inclusion criteria were being male between 16 and 20 years old, belonging to a youth soccer division of a professional club, and having a minimum of two years of HBS overload experience. The exclusion criteria were muscle injuries, tendon injuries, or fractures in the last three months that prevented the performance of the tests proposed in the study. Recruitment of participants and evaluations were performed between December 2023 and January 2024. Informed consent was provided on paper and signed before the start of the evaluations. Likewise, all participants were informed of the study objectives before signing the informed consent and assessments. The study’s protocol was approved by the Scientific-Ethical Committee of Universidad de Las Américas, Chile (registration number: CEC_FP_2023055). All study procedures were conducted under the Declaration of Helsinki (updated in 2013) and the ethical standards for exercise and sports [26].

### 2.3. Anthropometry

Body mass (kg) and fat mass were assessed with a digital weight (TANITA, model InnerScan BC-554^®^, Tokyo, Japan), and stature (m) was set with a stadiometer (SECA, model 700^®^, Hamburg, Germany), respecting the Frankfort plane and in maximum inspiration. Both body mass and stature were assessed in underwear. Body mass index (BMI) was calculated by dividing body mass in kilograms (kg) by stature in meters squared (m^2^).

### 2.4. Warm-Up Protocol

Participants performed a standardized 20 min warm-up, which included the following exercises: 1 × 10 repetitions (reps) of plantar flexion and dorsiflexion of the ankles, 1 × 10 reps of flexion and extension of the knees, 1 × 10 reps of flexion and extension of the hips, and 1 × 10 reps of flexion, extension, adduction, and abduction of the shoulders. After, they performed 6 min of jogging at 8 km·h^−1^. Subsequently, they added 2 × 10 s stretching of the legs, thighs, and hips muscle groups. For the 1RM test in the HBS, the participants performed the following specific warm-ups: 1 × 6 × 20 kg, 1 × 4 × 25 kg, 1 × 4 × 30 kg, and 1 × 2 × 40 kg. For the RSA test, the participants performed the following specific warm-up: 10 × 30 m skipping, 3 × 10 m heel-to-butt drill, four linear multi jumps + 10 m of the sprint, and four lateral multi jumps + 10 m of the sprint.

### 2.5. 1RM Test

The one repetition maximum (1RM) test in HBS was incremental, starting with 20 kg (barbell only) until failure or until the program projected the participant’s 1RM. Participants were asked to shift the weight vertically as fast as possible at each load performed. In addition, during the test, participants received verbal encouragement from the research team [27]. A Chrono Jump^®^ linear encoder and Chrono Jump version 1.4.6.0^®^ software (Barcelona, Spain) were used to evaluate 1RM in HBS [28].

### 2.6. RSA Test

Participants performed an RSA protocol before the four HBS repetitions to induce intra-session acute muscle fatigue—which can be evidenced during matches [3] or within the training itself [29]. The RSA protocol included 21 repetitions of 30 m with three direction changes [30,31]. The first change of direction was performed at 10 m (120° to the right), the second at 15 m (60° to the left), and the third and last at 20 m (120° to the right) (Figure 1). The 21 repetitions were divided into three sets of 7 repetitions with 25 s of passive recovery between repetitions and three 3 min pauses between sets. During the RSA assessments, time was measured using a Chrono Jump^®^ photocell and Chrono Jump version 1.4.6.0^®^ software (Barcelona, Spain). A gantry was used at the beginning (0 m) and another at the end of the run (30 m). The RSA was performed outdoors on a natural grass surface. During the test, players wore soccer boots and received verbal encouragement from the research team [27]. For the present study, the RSA was used to induce fatigue. Therefore, in addition to the total time of the 21 reps, the variable analyzed and included in the statistical analysis was the FI, calculated using the following equation [32]:

$$Fatigue\;Index=\left(\frac{mean\;time\;of\;21\;rep-best\;time}{best\;time}\right)\times 100$$

### 2.7. Muscle Power Development after Fatigue Induction

Following fatigue induction by RSA and after 10 min of rest, participants were asked to perform four repetitions of HBS with a load equivalent to 65% of 1RM. Participants were asked to shift the weight vertically at the highest possible speed for each load performed. In addition, during the test, the participants received verbal encouragement from the research team [27]. A Chrono Jump^®^ linear encoder and Chrono Jump version 1.4.6.0^®^ software (Barcelona, Spain) were used to evaluate the four HBS replicates [28]. In the statistical analysis, mean velocity (m·s^−1^), mean power (W), mean force (N), and RFD (N/s) were the variables used to quantify differences in performance before and after 14 days of creatine supplementation.

### 2.8. Supplementation

The EG was supplemented with Cr for 14 days. The daily dose was equivalent to 0.3 g·kg^−1^·day^−1^ [19]. Cr was dissolved in 200 mL of water and ingested orally between 18:00 and 20:00 h [33]. Participants were asked to ingest Cr with a carbohydrate-rich meal to optimize Cr absorption [34]. CG was supplemented with maltodextrin for 14 days (PL). The daily dose was equivalent to 0.3 g·kg^−1^·day^−1^. All participants (EG and CG) were asked to refrain from ingesting caffeine, yerba mate, alcoholic beverages, carbonated beverages, isotonic beverages, energy drinks, protein shakes, supplements defined as activators, or any substance that increased metabolism during the three weeks of the experiment. During the experiment, the diet was not administered. However, the participants were asked to continue their diet as prescribed by the Everton club. These dietary guidelines covered the nutritional needs associated with the energy demands of training and matches during the study.

### 2.9. Statistical Analysis

Descriptive data are presented as means and standard deviations (SDs). Considering there were 28 participants, the normal distribution of the data was confirmed by the Shapiro–Wilk test (*p* > 0.05). For the comparison between groups before applying the supplementation protocol, the *t*-test and Mann–Whitney test were employed for parametric and non-parametric samples, respectively (*p* ˂ 0.05). Age and muscle strength (1RM) were used for sample matching. Two-way repeated measures analysis of variance (two-way ANOVA) was applied with time (test vs. post-test) and group (EG vs. CG test vs. post-test) as factors to analyze the possible effects in vertical bar velocity, power, force, and RFD in HBS, FI and total time of RSA test. The Bonferroni post hoc test was employed to explore the differences between EG and CG in the test and post-test. The effect size (ES) was estimated by calculating the partial eta-squared (${ŋ}_{p}^{2}$): <0.01 small; >0.06: medium, and >0.14: large effect, respectively. A significance level of *p* < 0.05 was accepted for all statistical comparisons. All statistical analyses were performed with Prism version 10.2.0 for Windows^®^ software.

## 3. Results

At the time of the study, the 28 participants were 17.1 ± 0.9 years old, while anthropometric analysis showed a body mass of 68.5 ± 6.0 kg, a stature of 172.8 ± 6.6 cm, and a BMI of 19.8 ± 1.3 kg/m^2^. The age, anthropometric characteristics, basal physical performance, and the comparison between groups are shown in Table 1.

**Figure 2:**
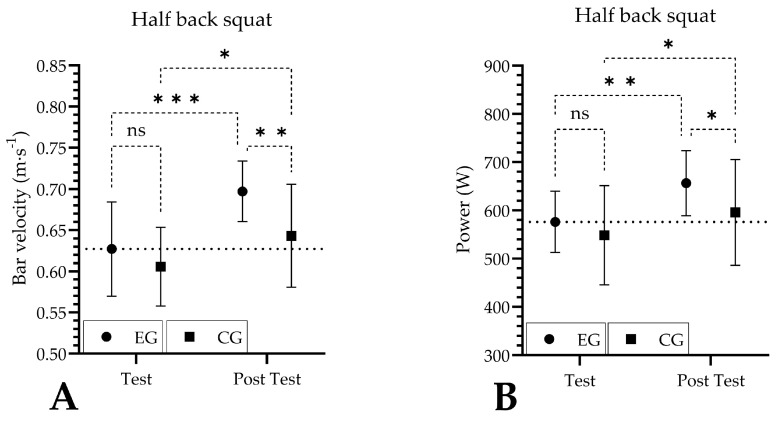

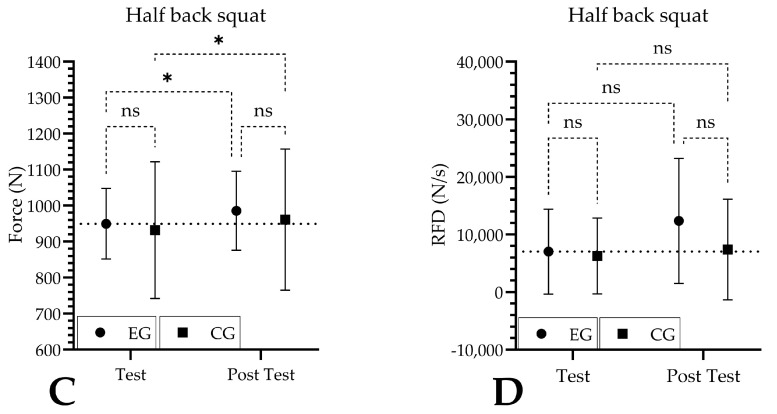
Outcomes in half back squat before and after creatine supplementation. (**A**) bar velocity in half back squat; (**B**) power in half back squat; (**C**) force in half back squat; (**D**) rate of force development in half back squat; CG: control group; EG: experimental group; m-s^−1^: meters per seconds; N; newton; N/s: newton per seconds; RFD: rate of force development; W: watts; ns: *p* > 0.05; *: *p* < 0.05; **: *p* < 0.01; ***: *p* < 0.001.

When analyzing bar velocity by both time and group, a significant increase was evident after 14 days of Cr supplementation (time: *p* = 0.0006; ${ŋ}_{p}^{2}$ = 0.22; group: *p* = 0.0431; ${ŋ}_{p}^{2}$ = 0.12, respectively). Time × group analysis for the same variable showed non-significant changes after 14 days of Cr supplementation (time × group: *p* = 0.0744, ${ŋ}_{p}^{2}$ = 0.02) (Figure 2A). The power generated in the HBS evidenced a significant increase for the time factor (time: *p* = 0.0006, ${ŋ}_{p}^{2}$ = 0.12), while the group and time × group factors showed non-significant changes (group: *p* = 0.16, ${ŋ}_{p}^{2}$ = 0.06; time × group: *p* = 0.17, ${ŋ}_{p}^{2}$ = 0.009, respectively) (Figure 2B). The force generated in HBS evidenced a significant increase for the time factor (time: *p* = 0.0013, ${ŋ}_{p}^{2}$ = 0.01), while the group and time × group factors showed non-significant changes (group: *p* = 0.70, ${ŋ}_{p}^{2}$ = 0.005; time × group: *p* = 0.64, ${ŋ}_{p}^{2}$ = 0.000, respectively) (Figure 2C). Finally, RFD evidenced a significant increase for the time factor (time: *p* = 0.0323, ${ŋ}_{p}^{2}$ = 0.03), while the group and time x group factors showed non-significant changes (group: *p* = 0.32, ${ŋ}_{p}^{2}$ = 0.02; time × group: *p* = 0.31, ${ŋ}_{p}^{2}$ = 0.01, respectively) (Figure 2D). A comparison of all variables in HBS between test and post-test for both EG and CG is reported in Figure 2.

**Figure 3:**
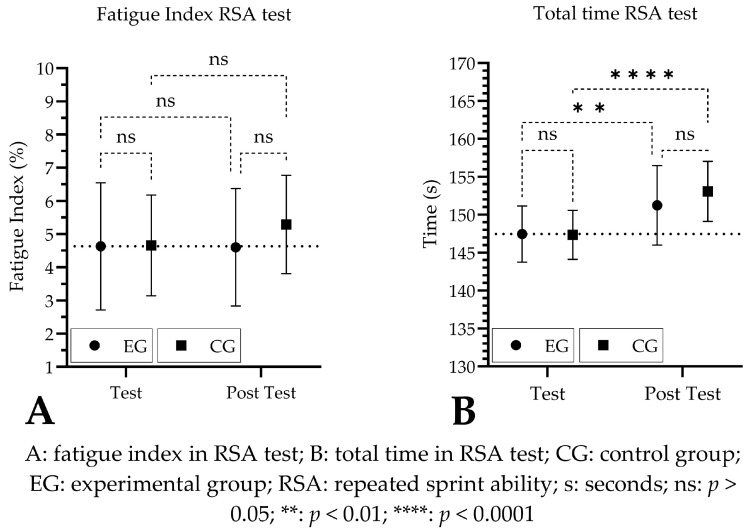
Outcomes in RSA test before and after creatine supplementation.

When analyzing the fatigue index in the RSA test by time, group, and time × group factor, no significant differences were observed after 14 days of Cr supplementation (time: *p* = 0.40, ${ŋ}_{p}^{2}$ = 0.009; group: *p* = 0.42, ${ŋ}_{p}^{2}$ = 0.012, time × group: *p* = 0.43, ${ŋ}_{p}^{2}$ = 0.010, respectively). When analyzing the differences in fatigue index between test and post-test, the EG decreased by 0.02% (95% CI of diff: −1.55 to 1.61), while the CG increased by 0.63% (95% CI of diff: −2.21 to 0.95). Figure 3A compares the fatigue index between the test and post-test for both EG and CG. The total time in the RSA evidenced a significant increase for the time factor (time: *p* < 0.0001, ${ŋ}_{p}^{2}$ = 0.266), while the group and time x group factors showed non-significant changes (group: *p* = 0.55, ${ŋ}_{p}^{2}$ = 0.012; time × group: *p* = 0.14, ${ŋ}_{p}^{2}$ = 0.015, respectively) (Figure 3B).

## 4. Discussion

The study determined the effect of a low dose of Cr administered orally for 14 days on muscle power after intra-session acute fatigue in young soccer players. The results of the study show that both the velocity of execution and the power generated in the HBS after the induction of acute intra-session fatigue through 21 repetitions of RSA were significantly higher in the group supplemented with Cr (EG) compared to the group supplemented with PL (CG). Consequently, using Cr increases physical performance in young soccer players, accepting the study hypothesis.

### 4.1. Muscle Strength and Power Production after Creatine Intake

Evidence shows that Cr ingestion benefits soccer players’ physical performance, especially anaerobic power [21]. In this context, Ostojic [35] evidenced significant changes in jump height after applying a protocol with 10 g of Cr ingested three times a day for seven days (*p* < 0.05). These results reinforce the present study’s findings, even more so if it is considered that the total amount of Cr ingested during the entire treatment, the characteristics of the sample, and the way of evaluating performance (vertical vector) were similar in both studies. In parallel, Yáñez-Silva et al. [22] determined the effect of 0.3 g·kg^−1^·day^−1^ Cr for 14 days on muscle power in young soccer players (the same low-dose protocol used in the present study). At the end of the study, the investigators reported an increase in peak and mean power in the Cr-supplemented group (*p* < 0.05) [22]. However, despite the similarity of the intake protocol used in both the Yáñez-Silva et al.’s [22] study and the present study, Yáñez-Silva et al.’s [22] investigation did not include exercises that induced intra-session acute fatigue before muscle power assessment. This intra-session acute fatigue induction was a crucial factor in the present study’s design, as it simulated the fatigue evidenced during matches [3] or within the training itself [29]. From a practical perspective, the changes observed in muscle power in the HBS during the present study acquire greater relevance, mainly due to the fatigue induction that resembles the accelerations, decelerations, changes in direction, sprints, and incomplete recoveries of a real match [3,29]. To the best of our knowledge [20,21], this is the first study to evaluate the execution velocity and powers generated in HBS after fatigue induction using an RSA protocol. In this scenario, supplementation with 0.3 g·kg^−1^·day^−1^ Cr for 14 days (low dose) effectively improved physical performance in young soccer players, ensuring intramuscular Cr saturation [33]. Likewise, it has been shown that lower-extremity explosive strength performance is a relevant factor for male soccer, so training should include exercises for its development [36]. Concerning the mechanisms that favor physical performance in short duration and high-intensity exercise, after Cr supplementation, the increase in intramuscular bioavailability of PCr, a compound that allows rapid ATP generation through the anaerobic PCr shuttle system, stands out [37]. In turn, this rapid ATP generation creates an environment conducive to cross-bridge maintenance, expanding the possibility of maintaining and increasing muscle strength and power, even more so in the later phases of exercise [6,38].

### 4.2. Fatigue Induction during High-Intensity Intermittent Exertion

An increased capacity for intermittent high-intensity efforts in soccer, including all categories, positively conditions sports performance [1]. However, the constant repetitions of intermittent high-intensity efforts evidenced during matches or training sessions [3,29] also induce muscle fatigue, negatively affecting physical performance [9]. In this context, it has been shown that the onset of fatigue is associated with increased myoplasmic inorganic phosphate [Pi] concentrations after exertion. This event affects cross-bridge function and inhibits muscle force production [6]. In parallel, other intracellular events that affect excitation–contraction coupling and induce fatigue are the progressive reduction in the ATP and PCr supply rate and the accumulation of lactate and hydrogen ions (H^+^) [7,37].

The literature suggests the use of Cr as a nutritional alternative to delay acute intra-session fatigue induced by high-intensity intermittent efforts in young soccer players, mainly because the breakdown of PCr (previously synthesized from Cr and Pi) produces approximately 10.3 kcal of free energy, potential energy that can be used to resynthesize ATP (ADP + PCr ↔ ATP + Cr) [23], with no evidence of adverse effects [14]. In this sense, Claudino et al. [39] examined the effects of Cr supplementation on lower-extremity muscle power in 14 elite soccer players, concluding that 20 g·day^−1^ Cr for a week, followed by 5 g·dau^−1^ for six weeks, prevents the decrease in lower-extremity muscle power in elite soccer players during progressive pre-season training. In the case of elite athletes, recommendations indicate that intake should be longer than 14 days, and if necessary, the daily dose should be increased to 19 g·day^−1^ for six weeks [19]. Returning to young soccer players and low doses of Cr, Mohebbi et al. [40] determined the effect of 20 g·day^−1^ Cr, for one week, on sprinting in young soccer players, concluding that this low dose of Cr increases performance in RSA (*p* = 0.001). Also, Williams et al. [41] determined the effect of 20 g·day^−1^ Cr, for one week, on physical performance in amateur soccer players, concluding that short-term supplementation (seven days) has no beneficial effect on physical tests simulating a 90 min soccer match (*p* ˃ 0.05). In these specific cases, with supplementation protocols of seven or fewer days, it is essential to review and analyze both group results and individual responses to this supplementation. Concerning the 14 days of supplementation used in the present study, it appears that a low dose of Cr positively affects the prevention of acute intra-session fatigue. Our findings show that EG decreased by 0.02% in the fatigue index during the 21 repetitions of 30 m (RSA), while GC increased by 0.63% in the same protocol, which justifies the use of a low dose of Cr to delay the onset of fatigue in young soccer players. Despite the above, the intake of a low dose in young soccer players requires further exploration. Likewise, it is important to analyze individual responses to this supplement (this can be found in the Supplementary Materials).

### 4.3. Limitations

During the development of this study, internal markers of intensity such as heart rate (HR) or post-exertion lactate were not included, nor were markers of muscle damage such as CK. We need this information to confirm that the participants have reached maximum intensity in each requested exercise. The intake of Cr contained in the daily diet of young soccer players was also not calculated. In the future, it will be necessary to determine the long-term changes in creatine supplementation. In addition, the next step would be to test another form of creatine, such as chelate [38]. Finally, it is essential to mention that a calculation of the total daily caloric expenditure of the participants should have been performed. Future research should include these variables and a review and analysis of the individual responses to the different doses of administration that appear in the scientific evidence.

## 5. Conclusions

At the end of this study, it was found that a low dose of Cr administered orally (0.3 g·kg^−1^·day^−1^) for 14 days after intra-session acute fatigue induction through 21 repetitions of RSA generates increases in physical performance in young soccer players, showing significant increases in both execution velocity and power generated in the HBS.

## 6. Practical Applications

A low dose of Cr for 14 days, administered orally, increased physical performance in young soccer players. However, before initiating this type of ergogenic aid in young soccer players, the following is suggested: (a) adequate supervision, (b) that young soccer players perform serious/competitive training, (c) that they consume a balanced diet that helps improve performance, (d) that they know the proper use of creatine, and (e) that they do not exceed the recommended doses [14].

## Figures and Tables

**Table 1:** Age, anthropometric characteristics, and basal physical performance.

	All	EG (n = 14)	CG (n = 14)	Mean Diff	95% CI of Diff	*t*	*p*-Value
Age (years) **	17.1 ± 0.9	17.2 ± 1.0	17.0 ± 0.9	−0.2 ± 0.3	−0.9 to 0.5	0.56	ns
Body mass (kg) *	68.5 ± 6.0	67.9 ± 5.3	69.1 ± 6.8	1.5 ± 2.3	−3.6 to 5.9	0.49	ns
Stature (m) *	1.72 ± 6.6	1.72 ± 6.8	1.73 ± 6.6	0.4 ± 2.5	−4.8 to 5.6	0.17	ns
BMI (kg/m^2^) *	19.8 ± 1.3	19.6 ± 1.1	19.9 ± 1.6	0.2 ± 0.5	−0.8 to 1.3	0.51	ns
Fat mass (kg) *	14.2 ± 3.2	13.2 ± 3.3	15.2 ± 2.8	2.0 ± 1.1	−0.4 to 4.4	1.7	ns
1RM (kg) *	133.5 ± 18.9	139.4 ± 16.4	135.7 ± 21.6	−3.7 ± 7.2	−18.6 to 11.2	0.51	ns

Kg: kilograms; kg/m^2^: kilograms per square meter; ns: not significant. *: For the comparison between groups, the *t*-test was employed. **: For the comparison between groups, the Mann–Whitney test was employed.

## Data Availability

Las contribuciones originales presentadas en el estudio se incluyen en el artículo/material suplementario, las consultas adicionales pueden dirigirse al autor o autores correspondientes.

## Supplementary Materials

Supplementary Material S1: https://doi.org/10.6084/m9.figshare.25556019; Supplementary Material S2: https://doi.org/10.6084/m9.figshare.25556022.

## References

[1] Kyles A., Oliver J.L., Cahill M.J., Lloyd R.S., Pedley J. (2023). Linear and Change of Direction Repeated Sprint Ability Tests: A Systematic Review. J. Strength Cond. Res..

[2] Sosa-Izquierdo J.J., Salas-Sánchez J., Latorre-Román P.Á. (2024). Caracterización Del Entrenamiento de La Fuerza En Futbolistas Profesionales y Semi-Profesionales de Las Ligas Españolas [Characterization of Strength Training in Professional and Semi-Professional Soccer Players in Spanish Leagues]. Retos.

[3] Nédélec M., McCall A., Carling C., Legall F., Berthoin S., Dupont G. (2012). Recovery in Soccer: Part I—Post-Match Fatigue and Time Course of Recovery. Sports Med..

[4] Ramírez J.M., Núñez V.M., Lancho C., Poblador M.S., Lancho J.L. (2015). Velocity-Based Training of Lower Limb to Improve Absolute and Relative Power Outputs in Concentric Phase of Half-Squat in Soccer Players. J. Strength Cond. Res..

[5] Thomakos P., Spyrou K., Katsikas C., Geladas N.D., Bogdanis G.C. (2023). Effects of Concurrent High-Intensity and Strength Training on Muscle Power and Aerobic Performance in Young Soccer Players during the Pre-Season. Sports.

[6] Allen D.G., Lamb G.D., Westerblad H. (2008). Skeletal Muscle Fatigue: Cellular Mechanisms. Physiol. Rev..

[7] Bigland-Ritchie B., Woods J.J. (1984). Changes in Muscle Contractile Properties and Neural Control during Human Muscular Fatigue. Muscle Nerve.

[8] Smith M.R., Coutts A.J., Merlini M., Deprez D., Lenoir M., Marcora S.M. (2016). Mental Fatigue Impairs Soccer-Specific Physical and Technical Performance. Med. Sci. Sports Exerc..

[9] Barte J.C.M., Nieuwenhuys A., Geurts S.A.E., Kompier M.A.J. (2020). Effects of Fatigue on Interception Decisions in Soccer. Int. J. Sport Exerc. Psychol..

[10] Nedelec M., Wisloff U., McCall A., Berthoin S., Dupont G. (2013). Recovery after an Intermittent Test. Int. J. Sports Med..

[11] Barbero-Álvarez J.C., Pedro R.E., Nakamura F.Y. (2013). Validity of a Repeated-Sprint Ability Test in Young Soccer Players. Sci. Sports.

[12] e Silva T.M., Abreu W.C., Pimenta E., da Silva S.F. (2023). Effects of Caffeine Supplementation on the Recovery of Professional Soccer Players. Muscles.

[13] Kreider R.B., Wilborn C.D., Taylor L., Campbell B., Almada A.L., Collins R., Cooke M., Earnest C.P., Greenwood M., Kalman D.S. (2010). ISSN Exercise and Sport Nutrition Review: Research and Recommendations. J. Int. Soc. Sports Nutr..

[14] Kerksick C.M., Wilborn C.D., Roberts M.D., Smith-Ryan A., Kleiner S.M., Jäger R., Collins R., Cooke M., Davis J.N., Galvan E. (2018). ISSN Exercise & Sports Nutrition Review Update: Research & Recommendations. J. Int. Soc. Sports Nutr..

[15] Trexler E.T., Smith-Ryan A.E. (2015). Creatine and Caffeine: Considerations for Concurrent Supplementation. Int. J. Sport. Nutr. Exerc. Metab..

[16] Jäger R., Purpura M., Shao A., Inoue T., Kreider R.B. (2011). Analysis of the Efficacy, Safety, and Regulatory Status of Novel Forms of Creatine. Amino Acids.

[17] Wax B., Kerksick C.M., Jagim A.R., Mayo J.J., Lyons B.C., Kreider R.B. (2021). Creatine for Exercise and Sports Performance, with Recovery Considerations for Healthy Populations. Nutrients.

[18] Antonio J., Candow D.G., Forbes S.C., Gualano B., Jagim A.R., Kreider R.B., Rawson E.S., Smith-Ryan A.E., VanDusseldorp T.A., Willoughby D.S. (2021). Common Questions and Misconceptions about Creatine Supplementation: What Does the Scientific Evidence Really Show?. J. Int. Soc. Sports Nutr..

[19] Kreider R.B., Kalman D.S., Antonio J., Ziegenfuss T.N., Wildman R., Collins R., Candow D.G., Kleiner S.M., Almada A.L., Lopez H.L. (2017). International Society of Sports Nutrition Position Stand: Safety and Efficacy of Creatine Supplementation in Exercise, Sport, and Medicine. J. Int. Soc. Sports Nutr..

[20] Guevara V.M., Montalva-Valenzuela F., Andrades-Ramírez O., Vargas J.J.N., Flores I., Castillo-Paredes A. (2024). Futbol y Creatina, Una Revisión Sistemática [Soccer and Creatine, a Systematic Review]. Retos.

[21] Mielgo-Ayuso J., Calleja-Gonzalez J., Marqués-Jiménez D., Caballero-García A., Córdova A., Fernández-Lázaro D. (2019). Effects of Creatine Supplementation on Athletic Performance in Soccer Players: A Systematic Review and Meta-Analysis. Nutrients.

[22] Yáñez-Silva A., Buzzachera C.F., Piçarro I.D.C., Januario R.S.B., Ferreira L.H.B., McAnulty S.R., Utter A.C., Souza-Junior T.P. (2017). Effect of Low Dose, Short-Term Creatine Supplementation on Muscle Power Output in Elite Youth Soccer Players. J. Int. Soc. Sports Nutr..

[23] Kreider R.B., Stout J.R. (2021). Creatine in Health and Disease. Nutrients.

[24] Ato M., López J.J., Benavente A. (2013). A Classification System for Research Designs in Psychology. An. Psicol..

[25] McKay A.K.A., Stellingwerff T., Smith E.S., Martin D.T., Mujika I., Goosey-Tolfrey V.L., Sheppard J., Burke L.M. (2022). Defining Training and Performance Caliber: A Participant Classification Framework. Int. J. Sports Physiol. Perform..

[26] Harriss D.J., MacSween A., Atkinson G. (2019). Ethical Standards in Sport and Exercise Science Research: 2020 Update. Int. J. Sports Med..

[27] McNair P.J., Depledge J., Brettkelly M., Stanley S.N. (1996). Verbal Encouragement: Effects on Maximum Effort Voluntary Muscle Action. Br. J. Sports Med..

[28] Sanchez-Medina L., Perez C.E., Gonzalez-Badillo J.J. (2010). Importance of the Propulsive Phase in Strength Assessment. Int. J. Sports Med..

[29] Chaouachi A., Manzi V., Wong D.P., Chaalali A., Laurencelle L., Chamari K., Castagna C. (2010). Intermittent Endurance and Repeated Sprint Ability in Soccer Players. J. Strength Cond. Res..

[30] Perroni F., Corvino M., Cignitti L., Minganti C. (2013). RSA Response to Preseason Training in Semiprofessional Soccer Players. Sport Sci. Health.

[31] Jorge G., Garrafoli M.T., Cal Abad C.C. (2020). Seasonal Repeated Sprint Ability With Change of Direction Variations in U17 and U20 Elite Brazilian Soccer Players: A Comparative Study. J. Strength Cond. Res..

[32] Oliver J.L. (2009). Is a Fatigue Index a Worthwhile Measure of Repeated Sprint Ability?. J. Sci. Med. Sport.

[33] Ribeiro F., Longobardi I., Perim P., Duarte B., Ferreira P., Gualano B., Roschel H., Saunders B. (2021). Timing of Creatine Supplementation around Exercise: A Real Concern?. Nutrients.

[34] Burke D.G., Chilibeck P.D., Parise G., Candow D.G., Mahoney D., Tarnopolsky M. (2003). Effect of Creatine and Weight Training on Muscle Creatine and Performance in Vegetarians. Med. Sci. Sports Exerc..

[35] Ostojic S.M. (2004). Creatine Supplementation in Young Soccer Players. Int. J. Sport Nutr. Exerc. Metab..

[36] Baldi M., Da Silva J.F., Buzzachera C.F., Castagna C., Guglielmo L.G. (2017). Repeated Sprint Ability in Soccer Players: Associations with Physiological and Neuromuscular Factors. J. Sports Med. Phys. Fit..

[37] Hall M., Manetta E., Tupper K. (2021). Creatine Supplementation: An Update. Curr. Sports Med. Rep..

[38] Zajac A., Golas A., Chycki J., Halz M., Michalczyk M.M. (2020). The Effects of Long-Term Magnesium Creatine Chelate Supplementation on Repeated Sprint Ability (RAST) in Elite Soccer Players. Nutrients.

[39] Claudino J.G., Mezêncio B., Amaral S., Zanetti V., Benatti F., Roschel H., Gualano B., Amadio A.C., Serrão J.C. (2014). Creatine Monohydrate Supplementation on Lower-Limb Muscle Power in Brazilian Elite Soccer Players. J. Int. Soc. Sports Nutr..

[40] Mohebbi H., Rahnama N., Moghadassi M., Ranjbar K. (2011). Effect of Creatine Supplementation on Sprint and Skill Performance in Young Soccer Players. Res. Sport Sci. Q..

[41] Williams J., Abt G., Kilding A.E. (2014). Effects of Creatine Monohydrate Supplementation on Simulated Soccer Performance. Int. J. Sports Physiol. Perform..

